# Individual-level social determinants of health and disparities in access to kidney transplant and waitlist mortality

**DOI:** 10.1371/journal.pone.0308407

**Published:** 2024-08-21

**Authors:** Tatenda G. Mupfudze, Alina Martinez, Samantha M. Noreen, Darren E. Stewart, Jesse D. Schold, Laura Cartwright

**Affiliations:** 1 Research Department, United Network for Organ Sharing, Richmond, Virginia, United States of America; 2 Departments of Surgery, NYU Langone Health, New York, New York, United States of America; 3 Departments of Surgery and Epidemiology, University of Colorado Anschutz Medical Campus, Aurora, Colorado, United States of America; Emory University, UNITED STATES OF AMERICA

## Abstract

**Background:**

Comprehensive, individual-level social determinants of health (SDOH) are not collected in national transplant registries, limiting research aimed at understanding the relationship between SDOH and waitlist outcomes among kidney transplant candidates.

**Methods:**

We merged Organ Procurement and Transplantation Network data with individual-level SDOH data from LexisNexis, a commercial data vendor, and conducted a competing risk analysis to determine the association between individual-level SDOH and the cumulative incidence of living donor kidney transplant (LDKT), deceased donor kidney transplant (DDKT), and waitlist mortality. We included adult kidney transplant candidates placed on the waiting list in 2020, followed through December 2023.

**Results:**

In multivariable analysis, having public insurance (Medicare or Medicaid), less than a college degree, and any type of derogatory record (liens, history of eviction, bankruptcy and/ felonies) were associated with lower likelihood of LDKT. Compared with patients with estimated individual annual incomes ≤ $30,000, patients with incomes ≥ $120,000 were more likely to receive a LDKT (sub distribution hazard ratio (sHR), 2.52; 95% confidence interval (CI), 2.03–3.12). Being on Medicare (sHR, 1.49; 95% CI, 1.42–1.57), having some college or technical school, or at most a high school diploma were associated with a higher likelihood of DDKT. Compared with patients with incomes ≤ $30,000, patients with incomes ≥ $120,000 were less likely to receive a DDKT (sHR, 0.60; 95% CI, 0.51–0.71). Lower individual annual income, having public insurance, at most a high school diploma, and a record of liens or eviction were associated with higher waitlist mortality.

**Conclusions:**

Patients with adverse individual-level SDOH were less likely to receive LDKT, more likely to receive DDKT, and had higher risk of waitlist mortality. Differential relationships between SDOH, access to LDKT, DDKT, and waitlist mortality suggest the need for targeted interventions aimed at decreasing waitlist mortality and increasing access to LDKT among patients with adverse SDOH.

## Introduction

An estimated 809,103 individuals in the United States (U.S.) currently live with End Stage Renal Disease (ESRD) [1]. Kidney transplantation is the optimal treatment option for ESRD [2]. However, less than 25,000 kidney transplant candidates receive a kidney transplant annually [3]. Social determinants of health (SDOH) have been shown to be associated with disparities in access to health care and health outcomes among patients with ESRD in single center studies [4–6]. However, comprehensive, individual-level SDOH are not collected in national transplant registries, limiting national research on the impact of SDOH on risk of mortality and access to living and deceased donor kidney transplant among transplant kidney candidates.

Several national registry studies have tried to get around this limitation by using patient Zip Code to link data from the Organ Procurement and Transplantation Network (OPTN), a national transplant registry, with public data sources including data from the U.S. Census Bureau’s American Community Survey [7, 8], the Centers for Disease Control [9], and the County Health Rankings project [10]. However, such studies are limited by the socioeconomic heterogeneity associated with the large population area covered by ZIP Code level data. Hence, such studies may underestimate the relationship between SDOH and waitlist outcomes.

To address this limitation, there is growing interest in the use of individual-level SDOH data from commercial data vendors to augment data from national transplant registries [6]. Prior studies outside of transplantation have demonstrated the utility of address data from commercial data vendors [11–17]. However, the use of individual-level SDOH from commercial data vendors has not been widely studied. The objective of this study was to apply the novel use of commercially derived, individual-level SDOH data to characterize the relationship between patient-level SDOH and three competing, waiting list outcomes: 1) access to living donor kidney transplant (LDKT), 2) access to deceased donor kidney transplant (DDKT), and 3) waitlist mortality, using a national cohort of waitlisted kidney candidates.

## Methods

### Data source and patient selection

**Fig 1:**
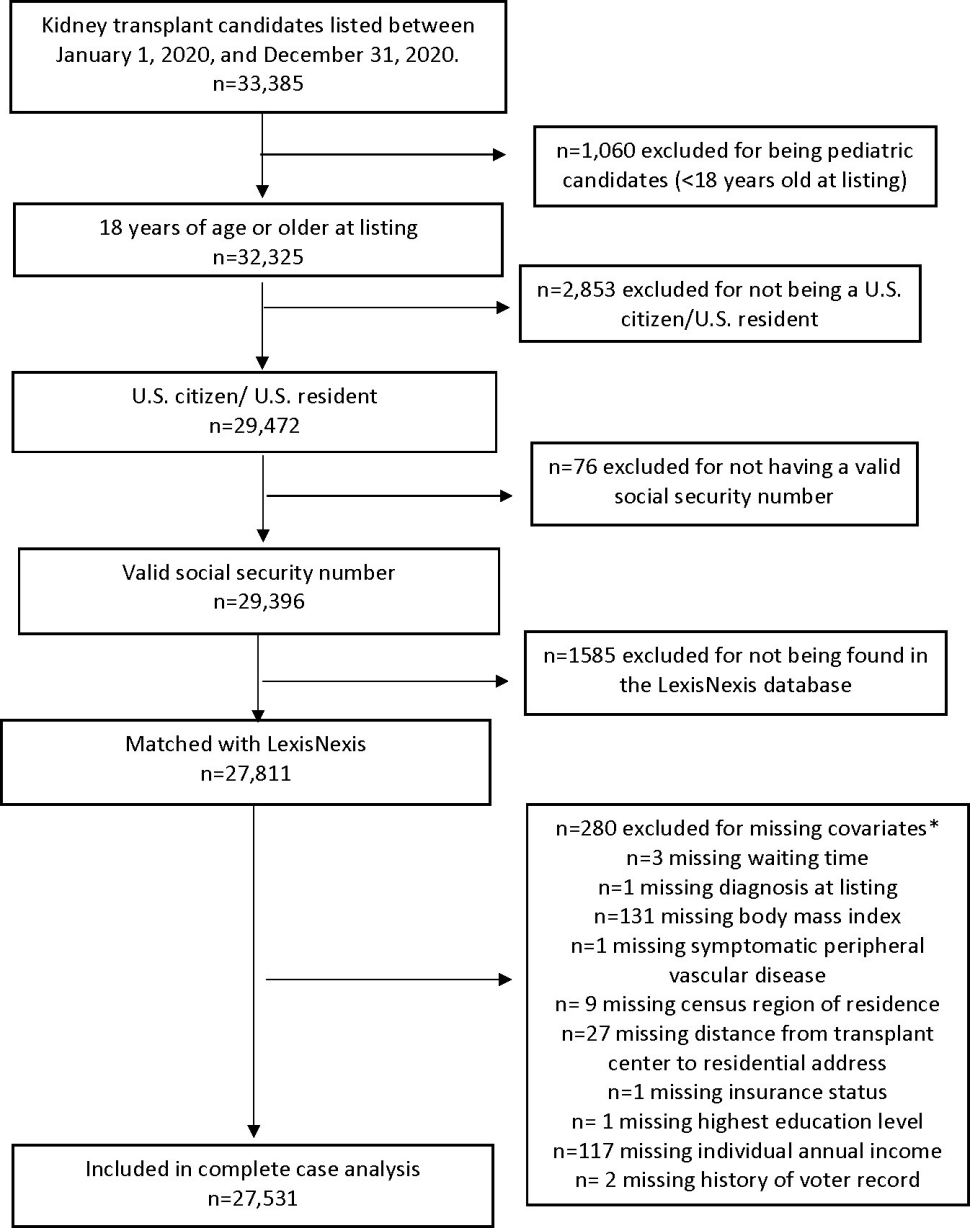
Kidney transplant candidates included and excluded from study cohort. *The number of excluded recipients summed by reason for exclusion is greater than total number excluded as a recipients may be missing more than one covariate.

This study used data reported to the Organ Procurement and Transplantation Network (OPTN). The OPTN data system includes data on all donors, wait-listed candidates, and transplant recipients in the U.S., submitted by the members of the OPTN. The Health Resources and Services Administration (HRSA), U.S. Department of Health and Human Services provides oversight to the activities of the OPTN contractor. OPTN data, including patient name, social security number (SSN), date of birth, age, sex, and home residence zip code were securely transmitted to LexisNexis and used to merge OPTN data with data from LexisNexis [11, 18]. Briefly, LexisNexis is a commercial data vendor that collects address and individual level-SDOH data for adults 18 years and older [18]. LexisNexis uses a proprietary algorithm to combine SDOH data for individuals from multiple databases, including real estate/tax assessor records, mortgage records, motor vehicle registrations, driver’s license records, federal and state tax liens, court filings (including bankruptcy, jury verdicts, settlements, and arbitrations), and voter registrations [11, 18]. Adult (≥ 18 years) kidney alone candidates added to the waitlist between January 1, 2020, and December 31, 2020, were included in this study (Fig 1). Kidney transplant candidates were included if they were U.S. citizens or U.S. residents and had a valid SSN. The first listing was included for candidates with multiple listings. All OPTN data analyzed are as of June 12, 2024, and are subject to change based on future data submission and correction.

### Outcomes and variables analyzed

This study had three primary outcomes 1) time to DDKT, 2) time to LDKT and 3) waitlist mortality, defined as death or delisting for too sick. Patient characteristics at listing, including age, sex, race/ethnicity, blood type, calculated panel reactive antibody (CPRA), body mass index (kg/m^2^), hypoalbuminemia (< 3.5 g/dL), peripheral vascular disease, time on dialysis, census region, insurance type, and highest level of education, were obtained from OPTN data. Race/ethnicity was categorized as non-Hispanic White, non-Hispanic Black, Hispanic/Latino, and non-Hispanic Other racial groups including non-Hispanic Asian/Asian-American, non-Hispanic American Indian/Alaska Native, non-Hispanic Multiracial, and non-Hispanic Native Hawaiian/Other Pacific Islander. Additional SDOH variables including estimated individual annual income, derogatory severity index, and voter registration records, were obtained from LexisNexis. The derogatory severity index, a measure of severity of derogatory record in the last 5 years, was categorized as no derogatory record, record contains liens, record contains bankruptcy, record contains eviction, and record contains felonies. For individuals with multiple derogatory records, the highest value is returned. Patient address obtained from LexisNexis was used to calculate distance to transplant center.

### Statistical analysis

Comparisons between groups were performed using the chi-square test and Fisher’s exact test for categorical variables and the Kruskal-Wallis test for non-normally distributed continuous data. We constructed competing risk models to examine associations between SDOH and DDKT, LDKT, and waitlist mortality. Removal for other reasons (removal for any reason other than DDKT, LDKT, or death on the waitlist) was treated as a competing risk. Competing risk models are appropriate when the occurrence of one outcome precludes the occurrence of the others, and research interest lies in understanding what factors are associated with the likelihood of each outcome occurring in a non-counterfactual (“real-world”) setting [19–21]. For example, the time to DDKT cannot be determined for candidates who received a LDKT; neither can the time to LDKT be measured for candidates who undergo a DDKT. Univariable and multivariable Fine-Gray models were constructed for each primary outcome. Variables were selected based on a priori hypothesis of a potential association with waitlist outcomes. Probability estimates at 3-years were compared using the Gray’s test for the cumulative incidence of each waitlist outcome. Pearson correlation coefficients were calculated to detect collinearity among SDOH variables, with a cut off of 0.7. Surviving patients were administratively censored at the end of the study (December 31, 2023). All analyses were performed using R version 3.5.0.

### Ethics statement

The Advarra Institutional Review Board reviewed and approved this study. Consent was waived as the study was deemed low risk by the IRB and the data were analyzed anonymously.

## Results

There were 29,472 U.S. citizen or permanent resident adult, kidney-alone transplant candidates listed between January 1, 2020, and December 31, 2020 (Fig 1). Over 94% were successfully linked to the LexisNexis database, while 5.6% (n = 1,661) were excluded due to inability to find a match including n = 76 without a valid SSN. Though matched candidates generally had similar characteristics as non-matched candidates, the median age was 3 years higher for matched compared to unmatched candidates, and the latter were more likely to reside in the South (53% versus 41%) (S1 Table).

After excluding 280 (1%) candidates with a missing covariate, a total of 27,531 patients were included in our study cohort: 33% (N = 9,167) received a DDKT; 15% (N = 4,044) received a LDKT; 15% (N = 4070) died while on the waiting list (Table 1). Most (66%) LDKT recipients were on private insurance compared with 36% of DDKT recipients and 33% of patients who died on the waitlist; 45% of LDKT had a college degree or higher compared with 27% of DDKT and 27% of patients who died on the waitlist. The median estimated individual annual income for LDKT recipients was $75,000 (IQR: $47,000-$96,000), versus $60,000 (IQR: $37,000-$78,000) for DDKT recipients, and $61,000 (IQR: $38,000-$79,000) for patients who died on the waitlist.

**Table 1:** Baseline characteristics of kidney transplant candidates listed in 2020 by waitlist outcome.

Patient Characteristics^a^	All Patients (N = 27531)	Living donor transplant (N = 4044)	Deceased donor transplant (N = 9167)	Death (or too sick to transplant) (N = 4070)	Removed for other reasons (N = 3596)	Still waiting on 12/31/23 (N = 6654)
**Age (years), median (IQR)**	55 (45–64)	52 (40–62)	55 (43–63)	61 (52–67)	54 (43–63)	55 (46–63)
**Sex**						
Male	16993 (62)	2556 (63)	5478 (60)	2660 (65)	2221 (62)	4078 (61)
Female	10538 (38)	1488 (37)	3689 (40)	1410 (35)	1375 (38)	2576 (39)
**Race/Ethnicity** ^**1**^						
Non-Hispanic White	12624 (46)	2717 (67)	3879 (42)	1887 (46)	1652 (46)	2489 (37)
Non-Hispanic Black	8302 (30)	473 (12)	3295 (36)	1246 (31)	1108 (31)	2180 (33)
Hispanic/Latino	4225 (15)	571 (14)	1289 (14)	617 (15)	537 (15)	1211 (18)
Non-Hispanic Other Race	2380 (9)	283 (7)	704 (8)	320 (8)	299 (8)	774 (12)
**Diagnosis at listing**						
Diabetes	10216 (37)	996 (25)	2932 (32)	2126 (52)	1547 (43)	2615 (39)
Glomerular Disease	4468 (16)	1063 (26)	1529 (17)	335 (8)	531 (15)	1010 (15)
Hypertensive Nephrosclerosis	4877 (18)	602 (15)	1832 (20)	642 (16)	601 (17)	1200 (18)
Tubular/Interstitial Disease	1055 (4)	174 (4)	328 (4)	143 (4)	116 (3)	294 (4)
Polycystic Kidney Disease	1897 (7)	533 (13)	566 (6)	107 (3)	242 (7)	449 (7)
Re-transplant/Graft Failure	1621 (6)	196 (5)	573 (6)	276 (7)	165 (5)	411 (6)
Other diagnosis	3397 (12)	480 (12)	1407 (15)	441 (11)	394 (11)	675 (10)
**Blood group**						
A	9326 (34)	1622 (40)	3587 (39)	1310 (32)	1167 (32)	1640 (25)
AB	1107 (4)	171 (4)	578 (6)	115 (3)	110 (3)	133 (2)
B	3966 (14)	530 (13)	1255 (14)	602 (15)	509 (14)	1070 (16)
O	13132 (48)	1721 (43)	3747 (41)	2043 (50)	1810 (50)	3811 (57)
**Body mass index (kg/m** ^**2**^ **)**						
<30	15537 (56)	2508 (62)	5275 (58)	2191 (54)	2110 (59)	3453 (52)
≥30-<35	7282 (26)	961 (24)	2318 (25)	1172 (29)	892 (25)	1939 (29)
≥35	4712 (17)	575 (14)	1574 (17)	707 (17)	594 (17)	1262 (19)
**CPRA at 4 weeks**						
0	19153 (70)	3134 (77)	5992 (65)	2814 (69)	2531 (70)	4682 (70)
1–80	6186 (22)	811 (20)	2106 (23)	929 (23)	809 (22)	1531 (23)
>80- <99	1539 (6)	85 (2)	775 (8)	232 (6)	172 (5)	275 (4)
99–100	653 (2)	14 (0)	294 (3)	95 (2)	84 (2)	166 (2)
**Hypoalbuminemia**						
No	22340 (81)	3396 (84)	7581 (83)	3014 (74)	2864 (80)	5485 (82)
Yes	4644 (17)	593 (15)	1487 (16)	952 (23)	635 (18)	977 (15)
Unknown	547 (2)	55 (1)	99 (1)	104 (3)	97 (3)	192 (3)
**Symptomatic Peripheral Vascular Disease**						
No	23820 (87)	3596 (89)	7909 (86)	3296 (81)	3176 (88)	5843 (88)
Yes	3468 (13)	425 (11)	1182 (13)	725 (18)	383 (11)	753 (11)
Unknown	243 (1)	23 (1)	76 (1)	49 (1)	37 (1)	58 (1)
**Time on Dialysis**						
Preemptive	7846 (28)	2034 (50)	1957 (21)	948 (23)	1055 (29)	1852 (28)
Less than 2 years	5322 (19)	1345 (33)	2095 (23)	733 (18)	719 (20)	430 (6)
2–4 years	7053 (26)	530 (13)	2291 (25)	1396 (34)	1139 (32)	1697 (26)
4+ years	7310 (27)	135 (3)	2824 (31)	993 (24)	683 (19)	2675 (40)
**Census region of residence**						
Northeast	5293 (19)	909 (22)	1406 (15)	810 (20)	671 (19)	1497 (22)
Midwest	5468 (20)	938 (23)	2121 (23)	760 (19)	701 (19)	948 (14)
West	5451 (20)	745 (18)	1653 (18)	723 (18)	590 (16)	1740 (26)
South	11319 (41)	1452 (36)	3987 (43)	1777 (44)	1634 (45)	2469 (37)
**Distance from transplant center (miles)** ^**2**^						
Quartile 1	6885 (25)	875 (22)	2465 (27)	961 (24)	802 (22)	1782 (27)
Quartile 2	6870 (25)	1145 (28)	2136 (23)	973 (24)	831 (23)	1785 (27)
Quartile 3	6907 (25)	1060 (26)	2309 (25)	1065 (26)	876 (24)	1597 (24)
Quartile 4	6869 (25)	964 (24)	2257 (25)	1071 (26)	1087 (30)	1490 (22)
**Insurance** ^**3**^						
Private insurance	12059 (44)	2660 (66)	3279 (36)	1330 (33)	1561 (43)	3229 (49)
Public insurance -Medicare	12622 (46)	1102 (27)	4982 (54)	2299 (56)	1635 (45)	2604 (39)
Public insurance -Medicaid	2174 (8)	202 (5)	695 (8)	309 (8)	300 (8)	668 (10)
Other insurance	676 (2)	80 (2)	211 (2)	132 (3)	100 (3)	153 (2)
**Highest education level**						
College Degree or higher	8502 (31)	1817 (45)	2477 (27)	1107 (27)	1149 (32)	1952 (29)
College or technical school	7512 (27)	1064 (26)	2504 (27)	1098 (27)	993 (28)	1853 (28)
High School or Less	10790 (39)	1052 (26)	3982 (43)	1758 (43)	1341 (37)	2657 (40)
Unknown	727 (3)	111 (3)	204 (2)	107 (3)	113 (3)	192 (3)
**Estimated individual annual income, in US dollars, median (IQR)**	63,000 (39,000–83,000)	75,000 (47,000–96,000)	60,000 (37,000–78,000)	61,000 (38,000–79,000)	63,000 (39,000–83,000)	65,000 (39,000–85,000)
**Estimated individual annual income (US dollars)**						
0–29,000	2104 (8)	146 (4)	859 (9)	338 (8)	281 (8)	480 (7)
≥30,000–49,000	9241 (34)	947 (23)	3413 (37)	1507 (37)	1227 (34)	2147 (32)
≥50,000–79,000	8541 (31)	1237 (31)	2835 (31)	1288 (32)	1102 (31)	2079 (31)
≥80,000–119,000	6660 (24)	1397 (35)	1860 (20)	854 (21)	850 (24)	1699 (26)
≥$120,000	985 (4)	317 (8)	200 (2)	83 (2)	136 (4)	249 (4)
**Derogatory public records**						
No derogatory record	14447 (52)	2730 (68)	4535 (49)	1890 (46)	1803 (50)	3489 (52)
Record contains bankruptcies	2137 (8)	242 (6)	653 (7)	362 (9)	285 (8)	595 (9)
Record contains filed liens	7085 (26)	807 (20)	2470 (27)	1198 (29)	957 (27)	1653 (25)
Record contains evictions	2966 (11)	211 (5)	1143 (12)	480 (12)	407 (11)	725 (11)
Record contains felonies	896 (3)	54 (1)	366 (4)	140 (3)	144 (4)	192 (3)
**History of voter record**						
No	15151 (55)	2104 (52)	4935 (54)	2185 (54)	1914 (53)	4013 (60)
Yes	12380 (45)	1940 (48)	4232 (46)	1885 (46)	1682 (47)	2641 (40)

Data are presented as median (IQR) for continuous variables and n (%) for categorical variables. CPRA, calculated panel reactive antibody; IQR, interquartile range

^1^Non-Hispanic Other race, non-Hispanic: non-Hispanic Asian (n = 1754); non-Hispanic American Indian/Alaska Native (n = 246); non-Hispanic Multiracial (n = 249); non-Hispanic Native Hawaiian/other Pacific Islander (n = 131).

^2^Distance from transplant center to residential address (miles) quartiles were distributed as ≤10.80, 10.81 to 27.69, 27.70 to 79.09, >79.10.

^3^Other insurance: Department of VA (n = 398), Other government (n = 252), CHIP (Children’s Health Insurance Program) (n = 4), Self (n = 16), Pending (n = 3), Donation (n = 2), Free Care (n = 1).

**Fig 2:**
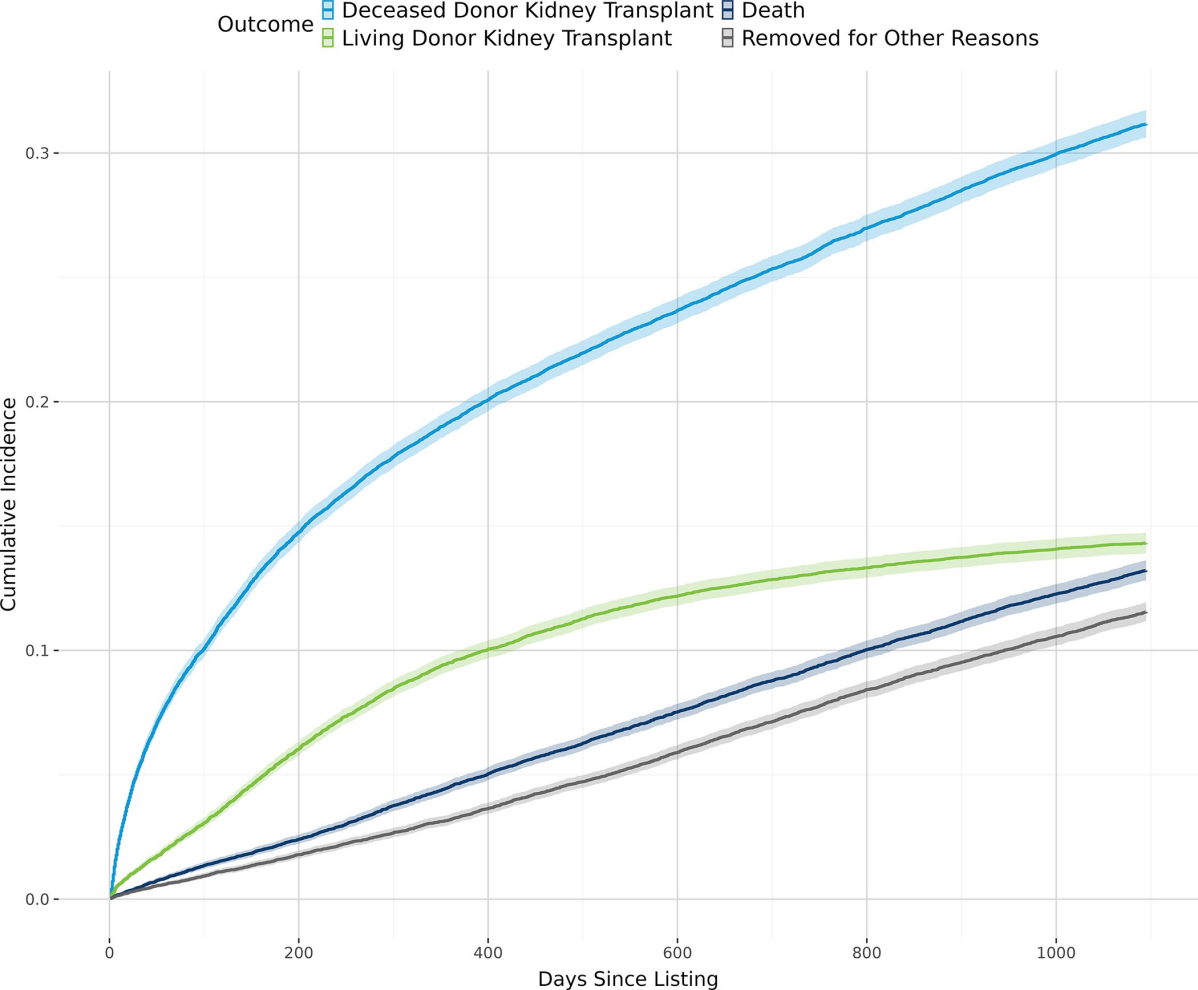
Unadjusted overall cumulative incidence probability of receiving a living donor kidney transplant, a deceased donor kidney transplant, death, or removal for other reasons, with 95% confidence interval.

**Fig 3:**
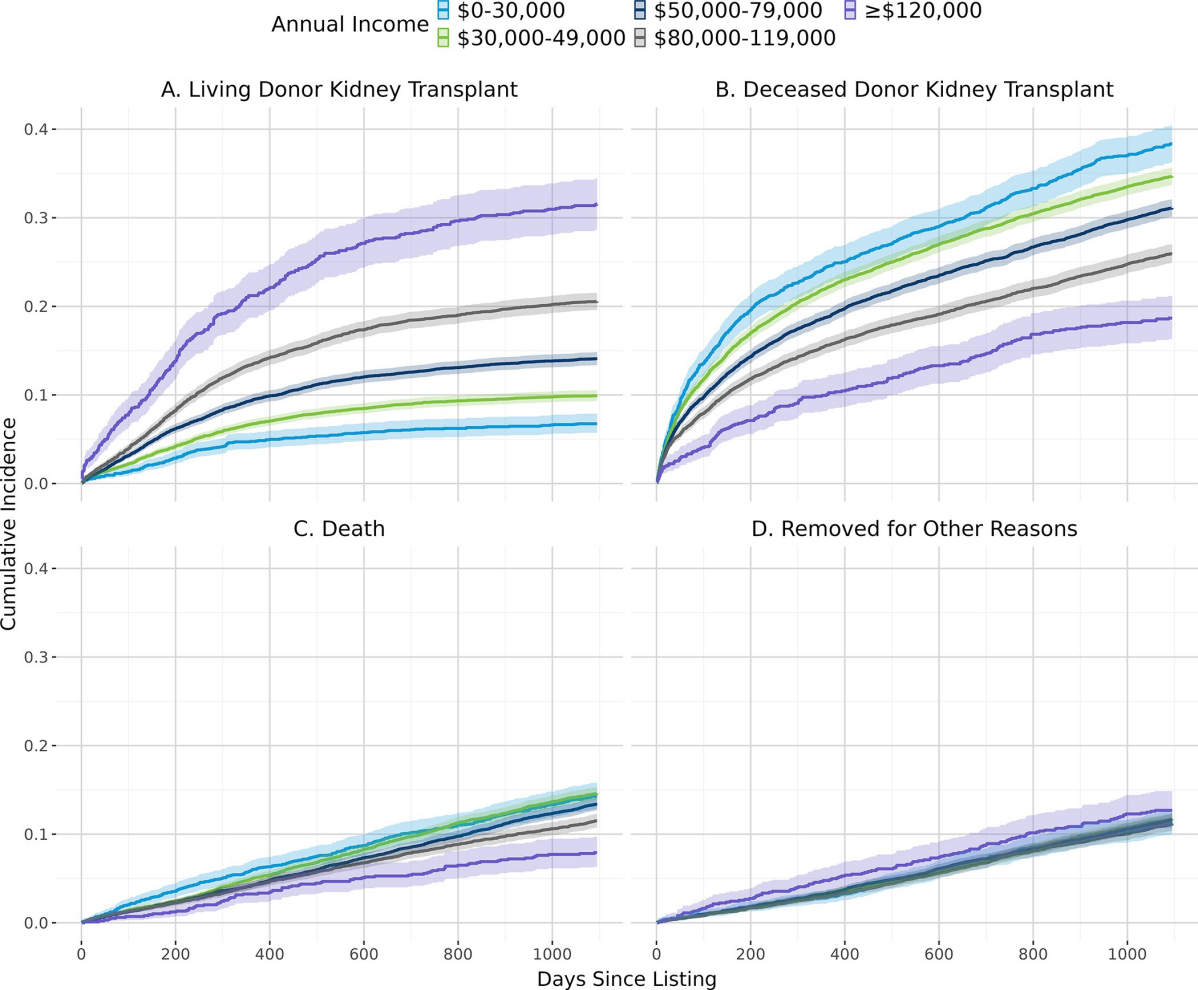
Unadjusted cumulative incidence probability of receiving a living donor kidney transplant (A), a deceased donor kidney transplant (B), death (C), or removal for other reasons (D) stratified by estimated individual annual income, with 95% confidence interval.

**Fig 4:**
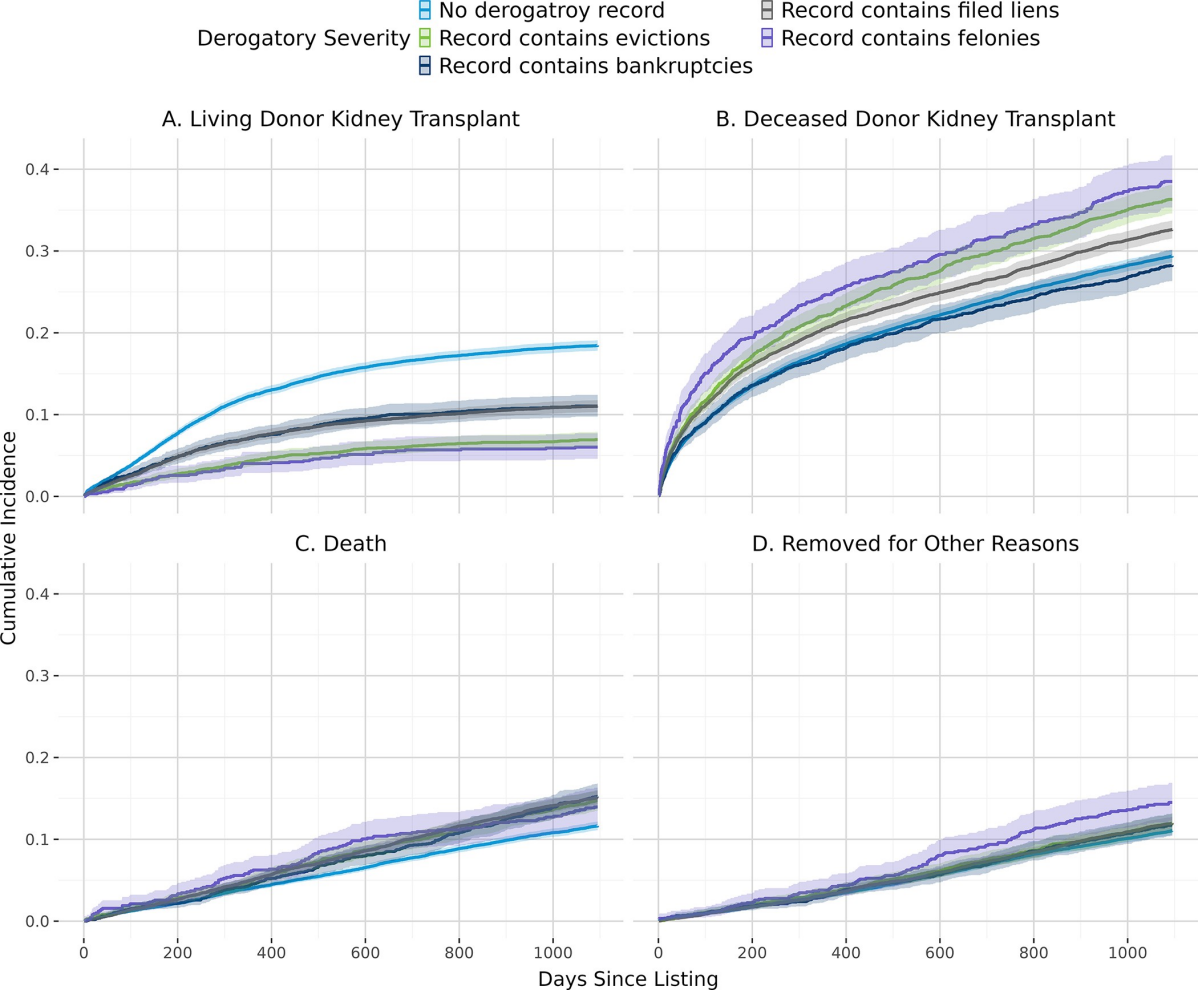
Unadjusted cumulative incidence probability of receiving a living donor kidney transplant (A), a deceased donor kidney transplant (B), death (C), or removal for other reasons (D) stratified by history of a derogatory record, with 95% confidence interval.

**Fig 5:**
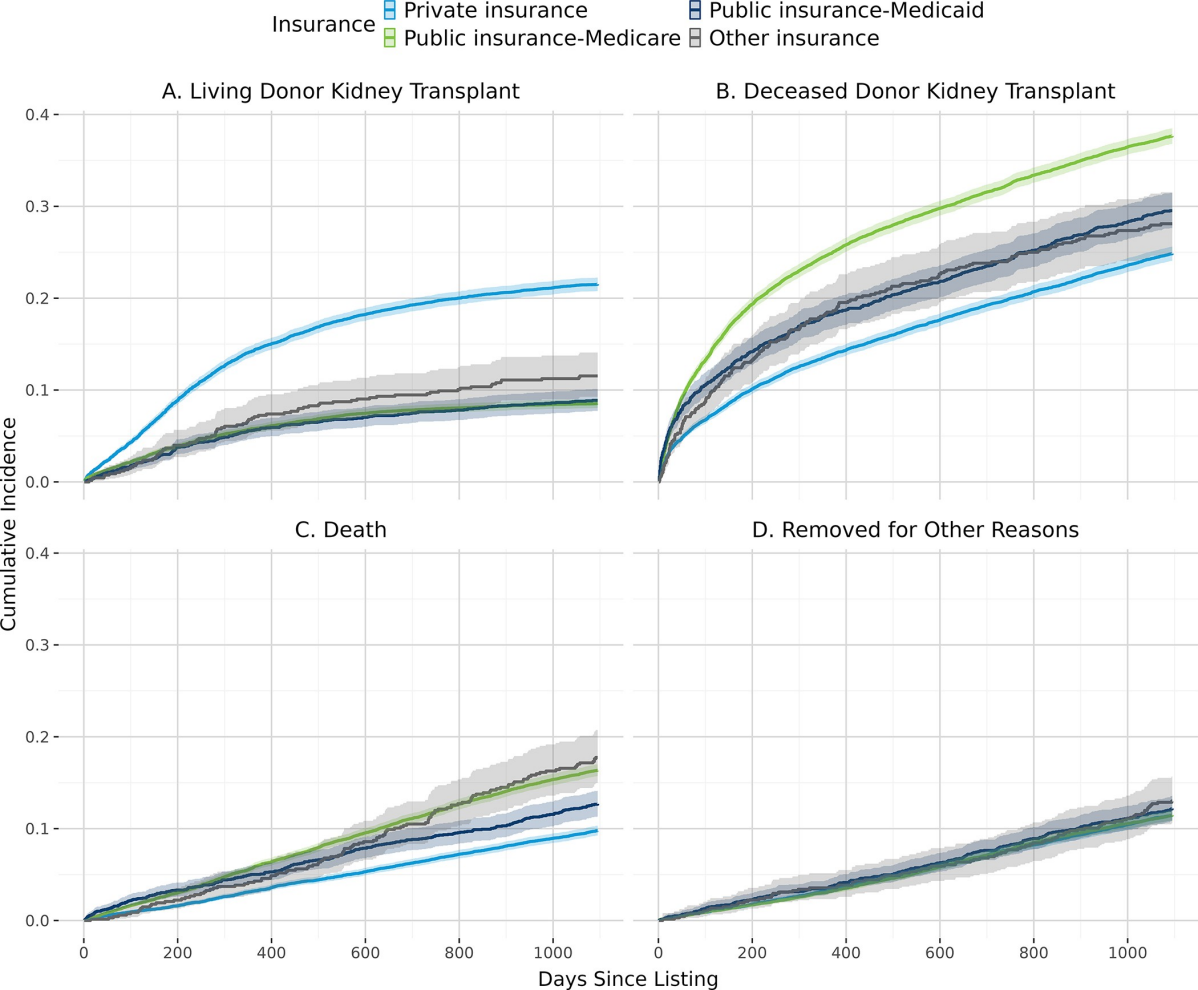
Unadjusted cumulative incidence probability of receiving a living donor kidney transplant (A), a deceased donor kidney transplant (B), death (C), or removal for other reasons (D) stratified by health insurance type, with 95% confidence interval.

**Fig 6:**
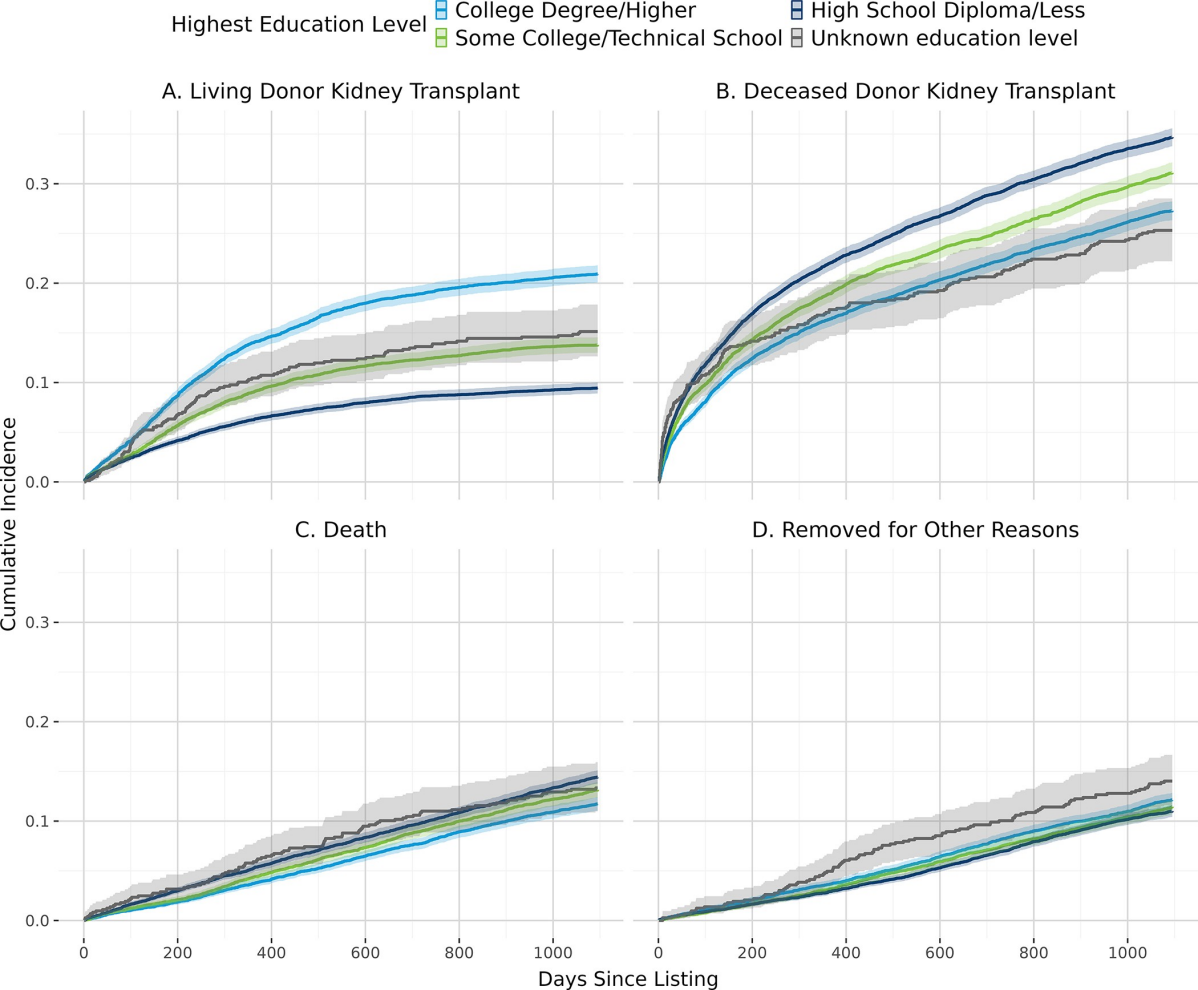
Unadjusted cumulative incidence probability of receiving a living donor kidney transplant (A), a deceased donor kidney transplant (B), death (C), or removal for other reasons (D) stratified by highest education level, with 95% confidence interval.

**Fig 7:**
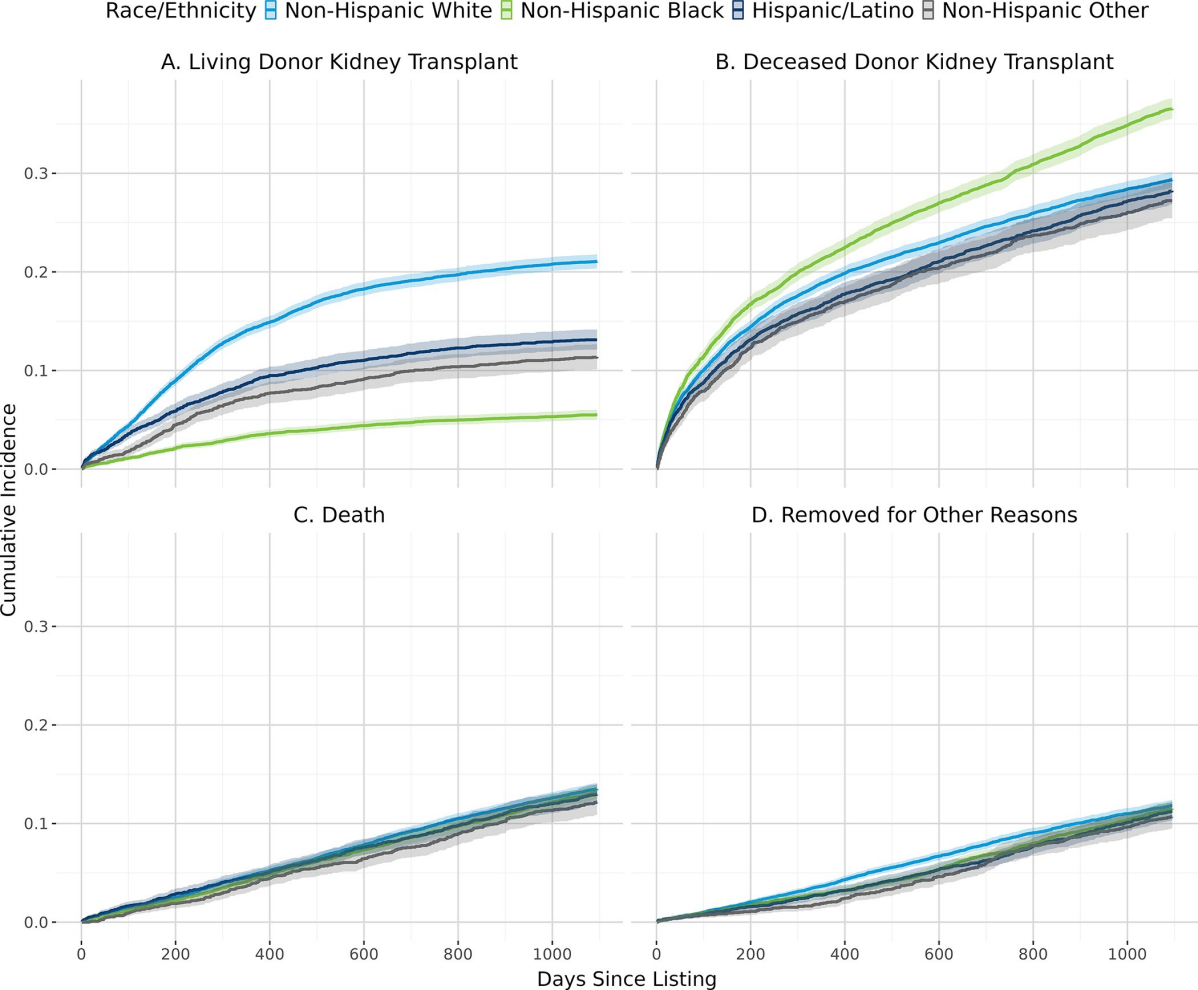
Unadjusted cumulative incidence probability of receiving a living donor kidney transplant (A), a deceased donor kidney transplant (B), death (C), or removal for other reasons (D) stratified by race/ethnicity, with 95% confidence interval.

Fig 2 shows the overall cumulative incidence of living donor kidney transplant, deceased donor kidney transplant, and death. In general, patients with lower estimated individual income (Fig 3), derogatory records (Fig 4), public insurance (Fig 5), and lower educational attainment (Fig 6) had lower cumulative incidence of LDKT, higher cumulative incidence of DDKT, and higher waitlist mortality. The 3-year cumulative incidence of receiving a LDKT for non-Hispanic White, non-Hispanic Black, Hispanic/Latino, and patients from non-Hispanic Other racial groups was 21%, 6%, 13%, and 11%, respectively (p<0.001; Fig 7A). The 3-year cumulative incidence of DDKT among Non-Hispanic White, Non-Hispanic Black, Hispanic/Latino, and Non-Hispanic Other race was 29%, 37%, 28%, and 27% respectively (p<0.001; Fig 7B). We found no significant difference in waitlist mortality by race/ethnicity (p = 0.25; Fig 7C).

### Multivariable associations between SDOH and waitlist outcomes

Table 2 shows the final multivariable model for each waitlist outcome. The dose response relationship between estimated individual annual income and likelihood of LDKT persisted in multivariable analysis; individuals with income ≥ $120,000 were more than twice as likely to receive a LDKT compared with those with income ≤ $30,000 (sub-distribution hazard ratio (sHR), 2.52; 95% confidence interval (CI), 2.03–3.12). Having public insurance, having less than a college degree or higher, and having any type of derogatory record (liens, history of eviction, bankruptcy and/ felonies) were associated with lower likelihood of LDKT. Compared with non-Hispanic White patients, non-Hispanic Black patients, and non-Hispanic Other racial groups had a lower likelihood of receiving a LDKT (sHR, 0.46; 95% CI, 0.42–0.52 and sHR, 0.60; 95% CI, 0.53–0.68, respectively).

**Table 2:** Multivariable Fine-Gray proportional sub-distribution hazards model for time from listing to receiving a living donor kidney transplant, a deceased donor kidney transplant, or death.

	Living donor transplant	Deceased donor transplant	Death (or too sick to transplant)
Patient Characteristics	sHR (95% CI)	sHR (95% CI)	sHR (95% CI)
**Age at listing**	0.98 (0.97, 0.98)	1.00 (1.00, 1.00)	1.04 (1.03, 1.04)
**Sex**			
Male	1(ref)	1(ref)	1(ref)
Female	0.92 (0.86, 0.99)	1.04 (0.99, 1.09)	0.87 (0.82, 0.93)
**Race/Ethnicity**			
Non-Hispanic White	1(ref)	1(ref)	1(ref)
Non-Hispanic Black	0.46 (0.42, 0.52)	1.22 (1.15, 1.29)	0.89 (0.81, 0.96)
Hispanic/Latino	1.02 (0.93, 1.13)	0.96 (0.90, 1.03)	0.90 (0.82, 1.00)
Non-Hispanic Other Race	0.60 (0.53, 0.68)	1.05 (0.96, 1.14)	0.93 (0.82, 1.06)
**Blood Type**			
A	1(ref)	1(ref)	1(ref)
AB	0.83 (0.70, 0.98)	1.57 (1.43, 1.73)	0.73 (0.60, 0.88)
B	0.98 (0.89, 1.09)	0.71 (0.66, 0.76)	1.14 (1.04, 1.26)
O	0.90 (0.84, 0.96)	0.65 (0.62, 0.68)	1.16 (1.08, 1.25)
**Diagnosis at listing**			
Diabetes	1(ref)	1(ref)	1(ref)
Glomerular Disease	1.62 (1.47, 1.78)	1.38 (1.29, 1.47)	0.52 (0.46, 0.58)
Hypertensive Nephrosclerosis	1.40 (1.26, 1.55)	1.35 (1.27, 1.43)	0.67 (0.62, 0.74)
Tubular/Interstitial Disease	0.94 (0.79, 1.11)	1.37 (1.22, 1.55)	0.72 (0.60, 0.85)
Polycystic Kidney Disease	1.52 (1.36, 1.69)	1.35 (1.23, 1.48)	0.35 (0.29, 0.43)
Re-transplant/Graft Failure	0.98 (0.84, 1.15)	1.13 (1.03, 1.24)	1.09 (0.95, 1.25)
Other diagnosis	0.85 (0.76, 0.95)	1.90 (1.77, 2.04)	0.75 (0.67, 0.84)
**Body mass index (kg/m** ^**2**^ **)**			
<30	1(ref)	1(ref)	1(ref)
≥30-<35	0.92 (0.85, 0.99)	0.95 (0.90, 0.99)	1.04 (0.97, 1.12)
≥35	0.86 (0.79, 0.95)	0.98 (0.92, 1.04)	1.06 (0.97, 1.16)
**CPRA at 4 weeks**			
0	1(ref)	1(ref)	1(ref)
1–80	0.90 (0.83, 0.97)	1.03 (0.98, 1.08)	1.05 (0.98, 1.14)
>80-<99	0.38 (0.30, 0.47)	1.55 (1.43, 1.68)	1.11 (0.97, 1.29)
99–100	0.20 (0.12, 0.34)	1.26 (1.10, 1.43)	1.16 (0.93, 1.44)
**Time on Dialysis**			
Preemptive	1(ref)	1(ref)	1(ref)
Less than 2 years	1.07 (1.00, 1.15)	1.77 (1.66, 1.89)	1.15 (1.04, 1.27)
2–4 years	0.33 (0.30, 0.36)	1.24 (1.16, 1.32)	1.45 (1.33, 1.58)
4+ years	0.10 (0.08, 0.12)	1.43 (1.34, 1.53)	0.93 (0.85, 1.03)
**Hypoalbuminemia**			
No	1(ref)	1(ref)	1(ref)
Yes	0.90 (0.82, 0.99)	0.96 (0.91, 1.02)	1.52 (1.41, 1.64)
Unknown	0.55 (0.42, 0.71)	0.54 (0.44, 0.66)	1.45 (1.19, 1.76)
**Symptomatic Peripheral Vascular Disease**			
No	1(ref)	1(ref)	1(ref)
Yes	1.26 (1.14, 1.41)	1.09 (1.02, 1.16)	1.16 (1.06, 1.26)
Unknown	0.61 (0.40, 0.93)	1.01 (0.79, 1.28)	1.50 (1.12, 2.02)
**Census region of residence**			
Northeast	1(ref)	1(ref)	1(ref)
Midwest	1.01 (0.92, 1.12)	1.59 (1.48, 1.70)	0.89 (0.80, 0.98)
West	0.74 (0.67, 0.82)	1.37 (1.26, 1.48)	0.97 (0.87, 1.08)
South	0.90 (0.82, 0.98)	1.36 (1.27, 1.45)	1.03 (0.94, 1.13)
**Distance from transplant center (miles)** ^**2**^			
Quartile 1	1(ref)	1(ref)	1(ref)
Quartile 2	1.08 (0.99, 1.18)	0.91 (0.85, 0.96)	1.09 (1.00, 1.20)
Quartile 3	1.08 (0.98, 1.18)	0.92 (0.87, 0.98)	1.14 (1.04, 1.24)
Quartile 4	1.10 (1.00, 1.21)	0.86 (0.81, 0.92)	1.12 (1.02, 1.22)
**Insurance** ^**3**^			
Private insurance	1(ref)	1(ref)	1(ref)
Public insurance -Medicare	0.78 (0.72, 0.84)	1.49 (1.42, 1.57)	1.30 (1.21, 1.40)
Public insurance -Medicaid	0.57 (0.49, 0.66)	1.07 (0.98, 1.16)	1.48 (1.31, 1.69)
Other insurance	0.76 (0.60, 0.94)	1.16 (1.01, 1.34)	1.48 (1.24, 1.78)
**Highest education level**			
College Degree or higher	1(ref)	1(ref)	1(ref)
College or technical school	0.90 (0.83, 0.98)	1.07 (1.01, 1.13)	1.07 (0.98, 1.17)
High School or Less	0.75 (0.69, 0.82)	1.19 (1.12, 1.25)	1.11 (1.02, 1.20)
Unknown	0.92 (0.76, 1.12)	1.01 (0.87, 1.17)	0.95 (0.77, 1.17)
**Estimated individual annual income (US dollars)**			
0–29,000	1(ref)	1(ref)	1(ref)
≥30,000–49,000	1.21 (1.01, 1.44)	0.98 (0.90, 1.06)	0.86 (0.77, 0.98)
≥50,000–79,000	1.48 (1.24, 1.77)	0.92 (0.85, 1.00)	0.79 (0.70, 0.90)
≥80,000–119,000	1.90 (1.59, 2.28)	0.82 (0.75, 0.89)	0.70 (0.61, 0.81)
≥$120,000	2.52 (2.03, 3.12)	0.60 (0.51, 0.71)	0.46 (0.35, 0.59)
**Derogatory public records**			
No derogatory record	1(ref)	1(ref)	1(ref)
Record contains bankruptcies	0.80 (0.70, 0.91)	0.97 (0.89, 1.05)	1.09 (0.97, 1.22)
Record contains filed liens	0.84 (0.77, 0.91)	1.09 (1.03, 1.15)	1.18 (1.10, 1.27)
Record contains evictions	0.69 (0.60, 0.80)	1.10 (1.02, 1.18)	1.33 (1.20, 1.48)
Record contains felonies	0.61 (0.46, 0.80)	1.15 (1.03, 1.29)	1.18 (0.99, 1.41)
**History of voter record**			
No	1(ref)	1(ref)	1(ref)
Yes	1.12 (1.05, 1.20)	1.08 (1.04, 1.13)	1.00 (0.94, 1.07)

^ǂ^Distance from transplant center to residential address (miles) quartiles were distributed as ≤10.80, 10.81 to 27.69, 27.70 to 79.09, >79.10.

Abbreviations: CPRA, calculated panel reactive antibody; sHR, Sub distribution Hazard Ratio.

An income ≥ $50,000 was associated with lower likelihood of DDKT. A record of liens or eviction in the past 5 years, being on Medicare (sHR, 1.49; 95% CI, 1.42–1.57), having some college or technical school (sHR, 1.07; 95% CI, 1.01–1.13), or at most a high school diploma (sHR, 1.19; 95% CI, 1.12–1.25), and having a voter registration record were associated with a higher likelihood of DDKT. Non-Hispanic Black patients were more likely to receive DDKT compared with non-Hispanic White patients (sHR, 1.22; 95% CI, 1.15–1.29).

Waitlist mortality was inversely associated with estimated individual annual income in a step wise fashion. We demonstrated that the likelihood of death on the waitlist was 66% lower among individuals with annual income ≥ $120,000 compared with those with annual income ≤ $30,000 (sHR, 0.46; 95% CI, 0.35–0.59). Having a record of liens or eviction, public or other insurance, and at most a high school diploma were associated with higher waitlist mortality. Compared to non-Hispanic White patients, non-Hispanic Black and Hispanic/Latino patients were less likely to die on the waitlist after adjusting for clinical and social risk factors (sHR, 0.89; 95% CI, 0.81–0.96 and sHR, 0.90; 95% CI, 0.82–1.00, respectively).

## Discussion

To our knowledge, this study is the first to apply the novel use of commercially derived individual-level SDOH to examine disparities in waitlist outcomes among a national cohort of kidney transplant candidates. In general, we demonstrated that among a recently listed patient cohort, patients with higher individual income, no or less severe derogatory records, higher education level, and private insurance were more likely to receive LDKT in lieu of DDKT. In contrast, adverse SDOH were associated with increased dependence on DDKT in place of LDKT, and increased risk of waitlist mortality. Our findings suggest that patients without adverse SDOH may be less dependent on access to DDKT. Hence, though having adverse SDOH was associated with increased likelihood of DDKT, this finding may be driven not by an inherent increase in DDKT access for patients with adverse SDOH, but rather by their markedly lower access to LDKT and our use of the competing risks analytical framework [20].

Our study adds to the literature by elucidating the differential relationships and interplay between individual-level SDOH and the competing outcomes of LDKT, DDKT, and risk of death on the waitlist. In addition to OPTN variables known to be associated with access to transplant and risk of death on the waitlist, we demonstrated that estimated individual annual income and absence of derogatory records–measures of individual economic stability–were associated with access to LDKT versus DDKT and waitlist mortality. Our findings on the relationship between lower individual annual income and lower access to LDKT are consistent with findings from single center and national registry studies using zip code-level measure of socioeconomic status (SES) [4, 8, 9] However, while prior studies have reported either no association between household income and access to DDKT [4]; or, have reported a weak, non-linear association between zip code-level measures of SES and access to DDKT [8], we found that individuals with adverse SDOH, including lower individual income and derogatory records, were more likely to receive DDKT compared with more advantaged patients. This finding does not necessarily imply an inherent disparity by SDOH in access to DDKT, but rather may merely reflect that candidates with fewer SDOH challenges have sharply elevated access to LDKT and are thus less dependent on waiting for a deceased donor organ.

There are also other plausible contributing explanations for the positive association between adverse SDOH and DDKT access. It is possible that the use of individual level-SDOH, in a national cohort of patients, minimized misclassification bias associated with zip code-level measures of SDOH, which may have biased inferences from prior studies. For example, prior studies have demonstrated 1) significant disagreement between individual- and area-level SDOH [22]; 2) stronger association between individual-level SDOH and health outcomes compared with area-level SDOH [22]. Hence, our study findings may highlight the utility of collecting individual-level SDOH, particularly measures of economic stability, including, but not limited to annual income in national registries.

It is also possible that the COVID-19 pandemic impacted the relationship between SDOH and access to DDKT. Given the impact of the COVID-19 pandemic on the U.S. transplant system [23], the study cohort reflects a select group of patients who were able to overcome barriers to listing during a pandemic. Hence, it may represent patients whose clinical and social risk factors expediated the need for a transplant. Consistent with our hypothesis, we found that patients with adverse SDOH had more clinical risk factors, including higher prevalence of diabetes, obesity, and longer time on dialysis, compared to more advantaged patients. We also demonstrated that patients with adverse SDOH experienced higher risk of waitlist mortality in tandem with higher likelihood of DDKT. Hence, the relationship between adverse SDOH and greater clinical risk factors simultaneously increases a candidate’s likelihood of DDKT and risk for mortality. As such, although patients with adverse SDOH who have overcome disparities in referral, evaluation, and waitlisting are more likely to receive DDKT, they are also at greater risk for waitlist mortality. It is also likely that lower access to LDKT may partially explain higher waitlist mortality among patients with adverse SDOH [24]. As noted earlier, disparities in COVID-19 related mortality likely strengthened the association between adverse SDOH and waitlist mortality. For example, a study by Schold et al found that lower educational attainment, Medicaid compared with private insurance, and zip code-level measures of SDOH (residential distress and residing in the most urban or most rural communities) were associated with higher rates of COVID-19 related mortality among kidney transplant candidates on the waitlist during the first 9 months of the pandemic [25]. Although not examined in prior work, it is also likely that individuals with adverse SDOH were disproportionately affected by the indirect effect of COVID-19 on access to and delivery of care.

## Limitations

Our study has several limitations. Most salient, although data from LexisNexis is derived from a combination of databases, some patients may be missing or have incomplete data, which may introduce selection and/or information bias, respectively [12]. And though the small portion of our sample that could not be successfully linked tended to be similar to the those that matched, the unmatched patients did differ statistically in terms of age, geography, and a few other factors. However, the potential to induce severe bias is likely limited since less than 6% of our study cohort could not be matched. It is also worth noting that due to the proprietary algorithm used to determine SDOH attributes we cannot validate or replicate data from commercial databases, including indices such as the derogatory severity index used in this study. However, it is encouraging that variables from LexisNexis were highly correlated with other SDOH, including insurance type and education level from OPTN data. Additionally, we did not account for unmeasured confounders, including variation in center specific practices. Although we did not test for interactions between SDOH, it is plausible the effect of multiple adverse SDOH, such as lower income, lower education level, and more severe derogatory record, may combine or interact, further exacerbating disparities in waitlist outcomes among candidates with adverse SDOH. Lastly, we only had access to individual-level SDOH for patients added to the waitlist in 2020 through our purchase agreement with LexisNexis. Although it is likely that the COVID-19 pandemic may have strengthened and/or attenuated the relationship between SDOH and waitlist outcomes, we are not able to explore the relationship between individual-level SDOH and waitlist outcomes prior to the pandemic in this analysis. In addition, though the median waiting time for a deceased donor kidney is slightly more than 4 years [26], our outcome window was limited to 3–4 years; hence, results could differ with a longer follow-up after listing.

## Conclusion

In summary, our study offers us a unique opportunity to examine the relationships between individual-level SDOH and kidney waitlist outcomes in the context of a global pandemic. Taken together, we demonstrated that patients without adverse individual-level SDOH were more likely to receive LDKT in lieu of DDKT. In contrast, patients with adverse SDOH were more reliant on DDKT versus LDKT and experienced higher waitlist mortality. Differential relationships between SDOH, access to LDKT, DDKT, and waitlist mortality suggest the need for targeted interventions aimed at decreasing waitlist mortality and increasing access to LDKT among patients with adverse SDOH. Additionally, future studies should examine the separate and joint effects of individual and area-level SDOH on kidney waitlist outcomes.

## Supporting information

S1 Table Baseline characteristics of kidney transplant candidates by LexisNexis database match results. (DOCX)
