# Supplementary material for: Individual-level social determinants of health and disparities in access to kidney transplant and waitlist mortality
Source: PLoS One. 2024 Aug 21;19(8):e0308407. doi: 10.1371/journal.pone.0308407 (PMC11338441; doi:10.1371/journal.pone.0308407)
Supplement: S1 Table — (DOCX) [file pone.0308407.s001.docx]

S1 Table 1. Baseline characteristics of kidney transplant candidates by LexisNexis database match results

|  | Matched with LexisNexis | | P-value |
| --- | --- | --- | --- |
| Patient Characteristics | Yes (n=27811) | No (n=1661)^¥^ |  |
| **Age (years)**, median (IQR) | 55.0 (45.0-64.0) | 52.0 (40.0-62.0) | <0.001 |
| **Sex** |  |  |  |
| Male | 17173 (62) | 1028 (62) | 0.993 |
| Female | 10638 (38) | 633 (38) |  |
| **Race/Ethnicity^1^** |  |  |  |
| Non-Hispanic White | 12758 (46) | 785 (47) | 0.275 |
| Non-Hispanic Black | 8379 (30) | 452 (27) |  |
| Hispanic/Latino | 4272 (15) | 262 (16) |  |
| Non-Hispanic Other Race | 2402 (9) | 162 (10) |  |
| **Diagnosis at listing** |  |  |  |
| Diabetes | 10292 (37) | 581 (35) | 0.58 |
| Glomerular Disease | 4511 (16) | 312 (19) |  |
| Hypertensive Nephrosclerosis | 4928 (18) | 299 (18) |  |
| Tubular/Interstitial Disease | 1068 (4) | 59 (4) |  |
| Polycystic Kidney Disease | 1912 (7) | 121 (7) |  |
| Re-transplant/Graft Failure | 1630 (6) | 100 (6) |  |
| Other | 3469 (12) | 189 (11) |  |
| **Blood group** |  |  |  |
| A | 9407 (34) | 515 (31) | 0.303 |
| AB | 1118 (4) | 70 (4) |  |
| B | 4013 (14) | 232 (14) |  |
| O | 13273 (48) | 844 (51) |  |
| **Body mass index*** |  |  |  |
| <30 | 15635 (56) | 978 (59) | 0.107 |
| ≥30-<35 | 7315 (26) | 430 (26) |  |
| ≥35 | 4730 (17) | 242 (15) |  |
| **CPRA at 4 weeks** |  |  |  |
| 0 | 19371 (70) | 1098 (66) | 0.076 |
| 1-80 | 6236 (22) | 429 (26) |  |
| >80- <99 | 1548 (6) | 98 (6) |  |
| 99-100 | 656 (2) | 36 (2) |  |
| **Hypoalbuminemia** |  |  |  |
| No | 22544 (81) | 1326 (80) | 0.762 |
| Yes | 4689 (17) | 295 (18) |  |
| Unknown | 578 (2) | 40 (2) |  |
| **Symptomatic Peripheral Vascular Disease*** |  |  |  |
| No | 24074 (87) | 1519 (91) | <0.001 |
| Yes | 3492 (13) | 134 (8) |  |
| Unknown | 244 (1) | 8 (0) |  |
| **Time on Dialysis** |  |  |  |
| Preemptive | 7953 (29) | 452 (27) | <0.001 |
| Less than 2 years | 5376 (19) | 408 (25) |  |
| 2-4 years | 7118 (26) | 449 (27) |  |
| 4+ years | 7364 (26) | 352 (21) |  |
| **Census Region of residence** |  |  |  |
| Northeast | 5378 (19) | 246 (15) | <0.001 |
| Midwest | 5504 (20) | 290 (17) |  |
| West | 5486 (20) | 238 (14) |  |
| South | 11434 (41) | 887 (53) |  |
| **Insurance^2*^** |  |  |  |
| Private insurance | 12180 (44) | 814 (49) | <0.001 |
| Public insurance -Medicare | 12725 (46) | 672 (40) |  |
| Public insurance -Medicaid | 2219 (8) | 105 (6) |  |
| Other insurance | 686 (2) | 70 (4) |  |
| **Highest education level*** |  |  |  |
| College Degree or higher | 8580 (31) | 576 (35) | <0.001 |
| College or technical school | 7570 (27) | 377 (23) |  |
| High School or Less | 10911 (39) | 640 (39) |  |
| Unknown | 749 (3) | 68 (4) |  |

Data are presented as median (IQR) for continuous variables and n (%) for categorical variables. CPRA, calculated panel reactive antibody; IQR, interquartile range

^¥^Included 76 patients who were not sent to LexisNexis because they were missing a valid social security number.

*Missing data: n=1 missing diagnosis at listing; n=142 missing BMI; n=1 missing symptomatic peripheral vascular disease; n= 9 missing census region of residence; n=1 missing insurance; n=1 missing highest education level.

^1^Non-Hispanic Other race: non-Hispanic Asian(n=1907); non-Hispanic American Indian/Alaska Native (n=257); non-Hispanic Multiracial (n =259); non-Hispanic Native Hawaiian/other Pacific Islander (n=141).

^2^Other insurance: Department of VA (n=398), Other government (n=252), CHIP (Children's Health Insurance Program) (n=4), Self (n=16), Pending (n=3), Donation (n=2), Free Care (n=1).
